# Shelterin-Like Proteins and Yku Inhibit Nucleolytic Processing of *Saccharomyces cerevisiae* Telomeres

**DOI:** 10.1371/journal.pgen.1000966

**Published:** 2010-05-27

**Authors:** Diego Bonetti, Michela Clerici, Savani Anbalagan, Marina Martina, Giovanna Lucchini, Maria Pia Longhese

**Affiliations:** Dipartimento di Biotecnologie e Bioscienze, Università di Milano-Bicocca, Milano, Italy; Brandeis University, United States of America

## Abstract

Eukaryotic cells distinguish their chromosome ends from accidental DNA double-strand breaks (DSBs) by packaging them into protective structures called telomeres that prevent DNA repair/recombination activities. Here we investigate the role of key telomeric proteins in protecting budding yeast telomeres from degradation. We show that the *Saccharomyces cerevisiae* shelterin-like proteins Rif1, Rif2, and Rap1 inhibit nucleolytic processing at both de novo and native telomeres during G1 and G2 cell cycle phases, with Rif2 and Rap1 showing the strongest effects. Also Yku prevents telomere resection in G1, independently of its role in non-homologous end joining. Yku and the shelterin-like proteins have additive effects in inhibiting DNA degradation at G1 de novo telomeres, where Yku plays the major role in preventing initiation, whereas Rif1, Rif2, and Rap1 act primarily by limiting extensive resection. In fact, exonucleolytic degradation of a de novo telomere is more efficient in *yku70Δ* than in *rif2Δ* G1 cells, but generation of ssDNA in Yku-lacking cells is limited to DNA regions close to the telomere tip. This limited processing is due to the inhibitory action of Rap1, Rif1, and Rif2, as their inactivation allows extensive telomere resection not only in wild-type but also in *yku70Δ* G1 cells. Finally, Rap1 and Rif2 prevent telomere degradation by inhibiting MRX access to telomeres, which are also protected from the Exo1 nuclease by Yku. Thus, chromosome end degradation is controlled by telomeric proteins that specifically inhibit the action of different nucleases.

## Author Summary

Telomeres are specialized nucleoprotein complexes that distinguish the natural ends of linear chromosomes from intrachromosomal double-strand breaks. In fact, telomeres are protected from DNA damage checkpoints, homologous recombination, or end-to-end fusions that normally promote repair of intrachromosomal DNA breaks. When chromosome end protection fails, dysfunctional telomeres are targeted by the DNA repair and recombination apparatus, whose outcomes range from the generation of chromosomal abnormalities, general hallmarks for human cancer cells, to permanent cell cycle arrest and cell death. While several studies address the consequences of telomere dysfunctions, the mechanisms by which telomere protection is achieved remain to be determined. Here, we investigate this issue by analyzing the role of evolutionarily conserved telomeric proteins in protecting budding yeast telomeres from degradation. We demonstrate that the key telomeric proteins Yku, Rap1, Rif1, and Rif2 inhibit telomere degradation by specifically preventing the action of different nucleases. As these proteins are functionally conserved between budding yeast and mammalian cells, they might also play critical roles in preventing telomere degradation in humans.

## Introduction

Intrachromosomal double-strand breaks (DSBs) elicit a DNA damage response, which comprises DNA repair pathways and surveillance mechanisms called DNA damage checkpoints. By contrast, telomeres are by definition stable and inert natural ends of linear chromosomes, as they are protected from checkpoints, as well as from homologous recombination (HR) or end-to-end fusions that normally promote repair of intrachromosomal DSBs (reviewed in [1]). Telomere basic structure is conserved among eukaryotes and consists of short tandem DNA repeats, which are G-rich in the strand containing the 3′ end (G-strand).

Although telomere ends are apparently shielded from being recognized as DSBs, they share important similarities with intrachromosomal DSBs. In fact, DSBs are resected to generate 3′-ended single-stranded DNA (ssDNA) tails, which channel their repair into HR. Similarly, the tips of human, mouse, ciliate, yeast and plant telomeres terminate with 3′ overhangs due to the protrusion of the G-strand over its complementary C-strand. Furthermore, several proteins such as the MRX complex, Sae2, Sgs1, Exo1 and Dna2 are required for generation of ssDNA at both telomeres and intrachromosomal DSBs, with Sae2 and MRX belonging to the same pathway, while the helicase Sgs1 acts in conjunction with the nuclease Dna2 [2]–[4]. Finally, both DSB and telomere resection is promoted by the activity of cyclin-dependent protein kinase Cdk1 [5]–[7], which phosphorylates Sae2 Ser267 [4], .

It is well known that ssDNA accumulation at DSBs invokes an ATR/Mec1-dependent DNA damage response when it exceeds a certain threshold [9]. Noteworthy, the single-stranded G-tails of budding yeast telomeres are short (about 10–15 nucleotides) for most of the cell cycle, and their length increases transiently at the time of telomere replication in late S phase [10]. As the nuclease requirements at DSBs and telomeres are similar [4], this finding suggests an inherent resistance of telomeric ends to exonuclease attack, which could contribute to avoid telomeres from being sensed as DNA damage. One report suggests that an elongating telomere formed at a TG-flanked DSB actually exerts an “anticheckpoint” effect on the non-TG-containing side of the break [11], though the origin of this checkpoint attenuation has been questioned [12].

In budding yeast, telomere protection is achieved through single- and double-stranded DNA binding proteins. In particular, the heterodimeric Yku complex (Yku70-Yku80) contributes to protect telomeres, as Yku lack causes shortened telomeres and Exo1-dependent accumulation of telomeric ssDNA [13]–[16], as well as checkpoint-mediated cell cycle arrest at elevated temperatures [15], [17]. Furthermore, Cdc13 inactivation leads to C-rich strand degradation, with subsequent accumulation of long ssDNA regions that extend into non-telomeric sequences [18]–[20]. Finally, the Rap1 protein, together with its interactors Rif1 and Rif2, binds telomeric double-stranded DNA repeats and inhibits both telomere fusions by non-homologous end joining (NHEJ) [21] and telomerase-dependent telomere elongation [22], [23]. The Rap1 C-terminal domain is sufficient for interaction with Rif1 and Rif2 [24]–[26] and is responsible for Rap1-mediated inhibition of both NHEJ and telomere elongation. In fact, deletion of Rap1 C-terminus causes both NHEJ-dependent telomeric fusions, due to the lack of Rif2 and Sir4 at telomeres [21], and an increase in telomere length, which is similar to the one observed when both Rif1 and Rif2 are lacking [26]. Proteins negatively regulating telomerase and NHEJ are found at telomeres also in other eukaryotes, such as fission yeast [27] and mammals, where they form a complex called shelterin that functionally recapitulates the Rap1-Rif1-Rif2 complex (reviewed in [28]).

Several studies address the consequences of telomere dysfunctions, while the mechanisms by which telomere protection is achieved remain to be determined. Here, we investigate this issue by analyzing the role of key telomeric proteins in protecting budding yeast telomeres from degradation. By using an inducible short telomere assay, we show that loss of Rif1 or Rif2, as well as deletion of Rap1 C-terminus, promotes C-rich strand degradation at an HO-derived telomere in G1 and enhances it in G2. The lack of Rap1 C-terminus or Rif2 shows the strongest effect at the induced short telomere and also causes ssDNA accumulation at native telomeres in cycling cells. Moreover, Yku prevents telomere resection in G1 at both native and HO-induced telomeres independently of its role in NHEJ. Resection of the HO-induced telomere in G1-arrested *yku70Δ* cells is restricted to the DNA regions closest to the telomeric tips, likely due to the action of Rap1, Rif1 and Rif2, whose inactivation extends telomere processing in *yku70Δ* G1 cells. Finally, ssDNA generation at both native and HO-induced telomeres requires Exo1 in *yku70Δ* G1 cells, whereas it depends primarily on MRX in both *rif2Δ* and *rap1ΔC* cells, where recruitment of the MRX subunit Mre11 to the HO-induced telomere is enhanced. Thus, while Yku protects telomeres from Exo1 action, the shelterin-like proteins prevent telomere degradation by inhibiting MRX loading onto telomeric ends.

## Results

### Rap1, Rif1, and Rif2 inhibit 3′ single-stranded overhang generation at a de novo telomere in both G1 and G2

**Figure 1:**
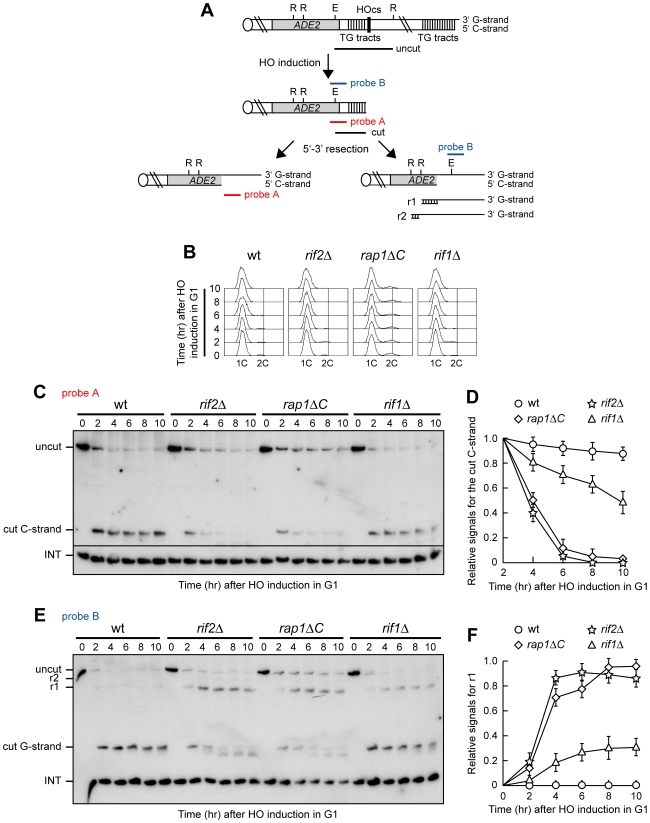
Rap1, Rif1, and Rif2 inhibit resection at a de novo telomere in G1. (A) The HO-induced telomere system. Galactose-induced HO endonuclease generates a single DSB at an HO cleavage site (HOcs) adjacent to an 81-bp TG repeat sequence (TG tracts) that is inserted at the *ADH4* locus on chromosome VII. RsaI- and EcoRV-digested genomic DNA was hybridized with two single-stranded riboprobes, which anneal to either the 5′ C-strand (probe A) or the 3′ G-strand (probe B) to a site located 212 bp from the HO cutting site. Both probes reveal an uncut 390 nt DNA fragment (uncut), which is converted by HO cleavage into a 166 nt fragment (cut) that can be detected by both probe A (5′ C-strand) and probe B (3′ G-strand). Degradation of the 5′ C-strand leads to disappearance of the probe A signal as resection proceeds beyond the hybridization region. Furthermore, it eliminates the cutting sites for the EcoRV (E) and RsaI (R) restriction enzymes, thus converting the 3′ cut G-strand into longer r1 (304 nt) and r2 (346 nt) DNA fragments detected by probe B. Both probes also detects a 138 nt fragment from the *ade2-101* locus on Chr. XV (INT), which serves as internal loading control. (B–F) HO expression was induced at time zero by galactose addition to α-factor-arrested wild type (YLL2599) and otherwise isogenic *rif2Δ*, *rap1ΔC* and *rif1Δ* cell cultures that were then kept arrested in G1. (B) FACS analysis of DNA content. (C) RsaI- and EcoRV-digested genomic DNA was hybridized with probe A. Degradation of the 5′ C-strand leads to the disappearance of the 166 nt signal (cut C-strand) generated by this probe. (D) Densitometric analysis. Plotted values are the mean value ±SD from three independent experiments as in (C). (E) The same RsaI- and EcoRV-digested genomic DNA analyzed in (C) was hybridized with probe B. Degradation of the 5′ C-strand leads to the conversion of the 3′ cut G-strand 166 nt fragment into the slower migrating r1 DNA fragment described in (A). (F) Densitometric analysis. Plotted values are the mean value ±SD from three independent experiments as in (E).

Nucleolytic degradation of telomeric ends is inhibited in G1, when Cdk1 (Cdc28/Clb in yeast) activity is low, whereas it occurs in G2/M cells, where Cdk1 activity is high [6], [7]. We investigated whether the shelterin-like proteins Rif1, Rif2 and Rap1 regulated 3′ overhang generation at *Saccharomyces cerevisiae* telomeres by examining the effects of their inactivation on telomeric ssDNA formation in both G1 and G2. We used an inducible short telomere assay (Figure 1A) [11], [29] that allows generation of a single short telomere without affecting the length of the other telomeres in the same cell. In this system, galactose-induced HO endonuclease generates a single DSB at an HO cleavage site adjacent to an 81-base pair TG repeat sequence that is inserted at the *ADH4* locus, 15 kb from the left telomere of chromosome VII (Figure 1A). After HO galactose-induction, the fragment distal to the break is lost, and, over time, the short telomeric “seed” sequence is elongated by telomerase [11],[29]. Length changes of either the 5′ C-strand or the 3′ G-strand of the newly created HO-induced telomere can be followed by using two single-stranded riboprobes (probes A and B in Figure 1) that detect the 5′ C-strand or the 3′ G-strand, respectively, by hybridizing to a DNA region spanning 212 bp from the HO site (Figure 1A).

HO was induced by galactose addition in G1-arrested *rif2Δ*, *rif1Δ* and *rap1ΔC* cells (Figure 1B), the latter lacking the Rap1 C-terminus (residues 670–807) that is sufficient for both telomere length regulation and Rap1 interaction with Rif1 and Rif2 [25], [26]. When the 5′ C-strand was analyzed with its complementary probe A in EcoRV and RsaI double-digested genomic DNA (Figure 1C), the predicted EcoRV-HO band (166 bp; cut C-strand) corresponding to the 5′ C-rich strand of the HO-induced telomere was detected in all cell cultures about 2 hours after HO induction. Consistent with the requirement of Cdk1 activity for telomere resection [6], [7], the C-strand signal was stable in G1-arrested wild type cells (Figure 1C and 1D). By contrast, it progressively decreased in both *rif2Δ* and *rap1ΔC* G1-arrested cells (Figure 1C and 1D), indicating that C-strand resection in these two mutants had proceeded beyond the hybridization region. C-strand degradation at the HO-derived telomere occurred also in *rif1Δ* cells, although less efficiently than in *rif2Δ* and *rap1ΔC* cells (Figure 1C and 1D). The decrease of single-stranded 5′ C-strand signal in all these mutants was due to DNA degradation and not to elongation by the coordinated action of telomerase and lagging strand DNA synthesis, as we observed a similar decrease also in *rif1Δ*, *rif2Δ* and *rap1ΔC* G1 cells lacking the catalytic subunit of telomerase (data not shown).

The 3′ G-strand of the HO-induced telomere was analyzed in the same DNA samples by using the G-strand complementary probe B (Figure 1E and 1F). Because EcoRV and RsaI do not cleave ssDNA, the 166 nt EcoRV-HO 3′ G-strand fragment is converted into slower migrating r1 and r2 DNA fragments as 5′ to 3′ resection proceeds beyond the EcoRV up to the two RsaI restriction sites located 304 and 346 bp, respectively, from the HO cutting site (Figure 1A). The amount of the predicted EcoRV-HO fragment (cut G-strand), which was constant in G1-arrested wild type cells, decreased over time in *rif1Δ*, *rif2Δ* and *rap1ΔC* cells that also showed r1 3′-ended resection products (Figure 1E and 1F), indicating that resection had proceeded beyond the EcoRV site towards the RsaI site located 304 bp from the HO cut. Again, the amount of the resection products was higher in *rif2Δ* and *rap1ΔC* cells than in *rif1Δ* cells (Figure 1E and 1F), indicating a stronger role for Rap1 and Rif2 in protecting telomeres from degradation in G1.

**Figure 2:**
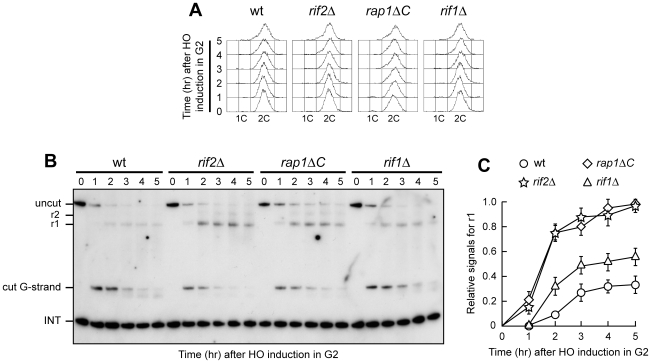
Rap1, Rif1, and Rif2 inhibit resection at a de novo telomere in G2. HO expression was induced at time zero by galactose addition to nocodazole-arrested wild type (YLL2599) and otherwise isogenic *rif2Δ*, *rap1ΔC* and *rif1Δ* cell cultures that were then kept arrested in G2. (A) FACS analysis of DNA content. (B) RsaI- and EcoRV-digested genomic DNA was hybridized with probe B as in Figure 1E. (C) Densitometric analysis. Plotted values are the mean value ±SD from three independent experiments as in (B).

Consistent with previous observations [29], 3′ G-strand length of the HO-induced telomere decreased by ∼10 nucleotides in both *rif2Δ* and *rap1ΔC* cells (Figure 1E). This very limited G-strand degradation was not specifically caused by the lack of Rif2 or Rap1, as it was detectable after HO induction also in G2-arrested wild type cells undergoing telomere resection (Figure 2B). A similar phenomenon has been described at intrachromosomal DSBs, where both the 5′ and the 3′ strands disappear with time in wild type cells after HO cleavage, and the 5′ strand is processed faster than the 3′ strand [9].

Telomere protection by the shelterin-like proteins occurred also outside G1, as shown by the analysis of 3′ single-stranded G-tail generation at the HO-induced telomere in G2-arrested *rif1Δ*, *rif2Δ* and *rap1ΔC* cells (Figure 2). As expected, r1 resection products were detectable in G2-arrested wild type cells, but their amount in these cells was significantly lower than in *rif2Δ*, *rap1ΔC* and *rif1Δ* cells (Figure 2B and 2C). Both *rif2Δ* and *rap1ΔC* G2 cells showed also some r2 resection products (Figure 2B), indicating that they allowed resection to proceed beyond the first RsaI site. The ∼10 nucleotides decrease in length of the 3′ G-strand occurring in G2-arrested wild type cells was not detectable in *rif2Δ* and *rap1ΔC* G2 cells (Figure 2B), likely because the 3′ G-strand in these two mutants was converted into longer r1 resection products much more efficiently than in wild type cells. Thus, Rif1, Rif2 and Rap1 inhibit degradation of the HO-induced telomere in both G1 and G2, with Rif2 and Rap1 playing the major role.

### Yku inhibits 3′ single-stranded overhang generation at a de novo telomere in G1

Yku lack accelerates 5′-to-3′ nucleolytic degradation of intrachromosomal DSBs in yeast cells with low Cdk1 activity [30]. This effect is partially due to NHEJ defects that might increase the time available to the resection machinery, as DSB processing is also increased in G1-arrested cells lacking the NHEJ DNA ligase IV (Dnl4/Lig4), although to a lesser extent than in *yku70Δ* cells [30].

**Figure 3:**
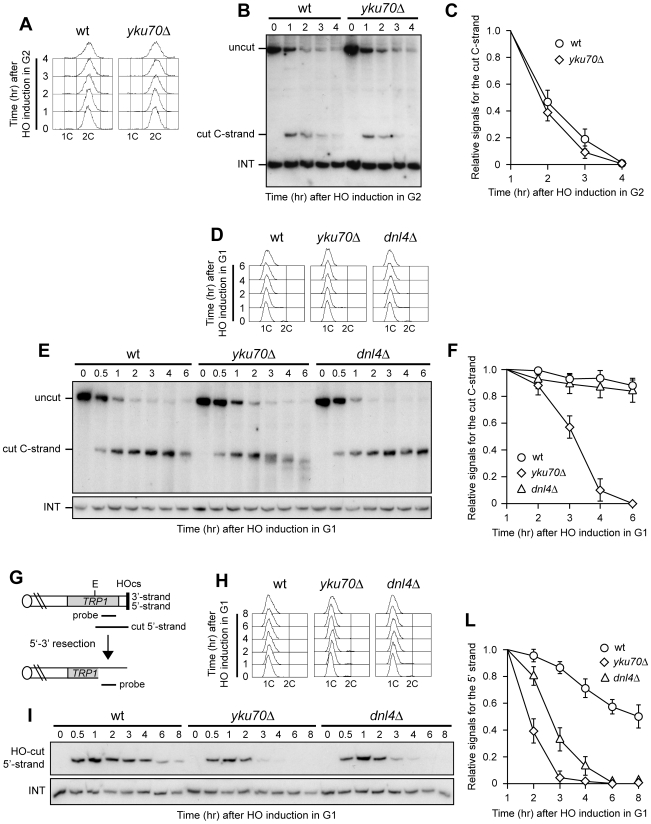
Yku inhibits resection at a de novo telomere specifically in G1. (A–C) HO expression was induced at time zero by galactose addition to nocodazole-arrested wild type (YLL2599) and otherwise isogenic *yku70Δ* cell cultures that were then kept arrested in G2. (A) FACS analysis of DNA content. (B) RsaI- and EcoRV-digested genomic DNA was hybridized with probe A as described in Figure 1C. (C) Densitometric analysis. Plotted values are the mean value ±SD from three independent experiments as in (B). (D–F) HO expression was induced at time zero by galactose addition to α-factor-arrested wild type (YLL2599) and otherwise isogenic *yku70Δ* and *dnl4Δ* cell cultures that were then kept arrested in G1. (D) FACS analysis of DNA content. (E) RsaI-digested genomic DNA was hybridized with the single-stranded riboprobe A described in Figure 1A, which anneals to the 5′ C-strand and reveals an uncut 460 nt DNA fragment (uncut). After HO cleavage, this fragment is converted into a 304 nt fragment (cut) detected by the same probe (cut C-strand). (F) Densitometric analysis. Plotted values are the mean value ±SD from three independent experiments as in (E). (G) The system used to generate an HO-induced DSB. Hybridization of EcoRV-digested genomic DNA with a probe that anneals to the 5′ strand to a site located 215 nt from the HO cutting site reveals a 430 nt HO-cut 5′-strand fragment. Loss of the 5′ strand beyond the hybridization region leads to disappearance of the signal generated by the probe. (H–L) HO expression was induced at time zero by galactose addition to α-factor-arrested wild type (YLL2600) and otherwise isogenic *yku70Δ* and *dnl4Δ* cells, all carrying the system in (G). Cells were then kept arrested in G1. (H) FACS analysis of DNA content. (I) EcoRV-digested genomic DNA was hybridized with the probe indicated in (G). The INT band, corresponding to a chromosome IV sequence, serves as internal loading control. (L) Densitometric analysis. Plotted values are the mean value ±SD from three independent experiments as in (I).

We investigated the possible role of Yku and/or Dnl4 in preventing telomere resection by analyzing the effect of their loss on the kinetics of 5′ C-strand degradation at the HO-induced telomere in both G1 and G2. We also evaluated how the lack of Yku and Dnl4 influenced 5′-strand degradation at an HO-induced DSB lacking the terminal TG repeats (Figure 3G) [11], in order to highlight possible differences in the regulation of DNA degradation at DSBs versus telomeres. Similar to what was found at intrachromosomal DSBs [30], Yku absence did not enhance processing of the HO-induced telomere in G2, as G2-arrested wild type and *yku70Δ* cells (Figure 3A) displayed very similar kinetics of 5′ C-strand degradation (Figure 3B and 3C). By contrast, the amount of 5′ C-strand of the HO-induced telomere decreased in G1-arrested *yku70Δ* cells, while it remained constant in both wild type and *dnl4Δ* cells under the same conditions (Figure 3D–3F). As expected [30], the 5′-strand at the HO-induced DSB lacking the TG repeats (Figure 3G) was degraded much more efficiently in both G1-arrested *yku70Δ* and *dnl4Δ* cells than in wild type, with *yku70Δ* cells showing the strongest effect (Figure 3H–3L). Thus, Dnl4 does not block telomere resection in G1, whereas Yku does, indicating that the role of Yku in telomere protection is not related to its NHEJ function. This finding also highlights differences in the regulation of nucleolytic processing at DSBs versus telomeres.

### Rif1, Rif2, and Rap1 limit resection at a de novo telomere in *yku70Δ* G1 cells

**Figure 4:**
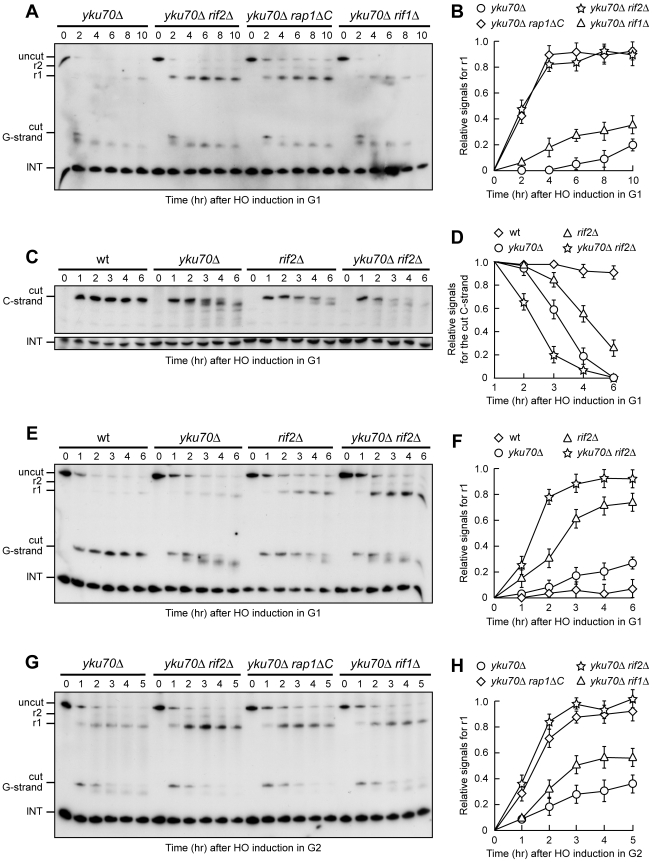
Rif2 and Rap1 inactivation enhances resection at a de novo telomere in *yku70Δ* cells. (A–F) HO expression was induced at time zero by galactose addition to α-factor-arrested cells with the indicated genotypes that were then kept arrested in G1. (A) RsaI- and EcoRV-digested genomic DNA was hybridized with probe B as in Figure 1E. (B) Densitometric analysis. Plotted values are the mean value ±SD from three independent experiments as in (A). (C) RsaI-digested genomic DNA was hybridized with probe A as in Figure 3E. (D) Densitometric analysis. Plotted values are the mean value ±SD from three independent experiments as in (C). (E) RsaI- and EcoRV-digested genomic DNA was hybridized with probe B as in Figure 1E. (F) Densitometric analysis. Plotted values are the mean value ±SD from three independent experiments as in (E). (G,H) HO expression was induced at time zero by galactose addition to nocodazole-arrested cells with the indicated genotypes that were then kept arrested in G2. (G) RsaI- and EcoRV-digested genomic DNA was hybridized with probe B as in Figure 1E. (H) Densitometric analysis. Plotted values are the mean value ±SD from three independent experiments as in (G).

Interestingly, G1-arrested *yku70Δ* cells converted the 5′ C-strand fragment of the HO-induced telomere into discrete smaller DNA fragments (Figure 3E), suggesting that C-strand degradation under these conditions is limited to the terminal part. In order to confirm this observation, we monitored the 3′ G-strand of the HO-induced telomere in *yku70Δ* cells. As shown in Figure 4A and 4B, the 3′ cut G-strand was not converted into the longer resection products r1 and r2 in G1-arrested y*ku70Δ* cells. Therefore, exonucleolytic degradation did not proceed beyond the EcoRV site located 166 bp from the HO site.

Thus, other proteins might limit resection of the HO-induced telomere in G1 even in the absence of Yku70, and the shelterin-like proteins appear to exert this effect. In fact, 3′-ended r1 resection products were clearly detectable in G1-arrested *yku70Δ rif2Δ*, *yku70Δ rap1ΔC* and, although to a lesser extent, *yku70Δ rif1Δ* cells (Figure 4A and 4B). Furthermore, the smaller C-strand fragments that accumulated in G1-arrested *yku70Δ* cells were only slightly detectable in similarly treated *yku70Δ rif2Δ* (Figure 4C) and *yku70Δ rap1ΔC* cells (data not shown), indicating that 5′ C-strand degradation in these cells had proceeded beyond 166 bp from the HO site. Thus, Rap1, Rif2 and, to a lesser extent, Rif1 limit telomeric ssDNA generation in G1 cells lacking Yku.

Notably, although telomere resection in *yku70Δ* G1 cells was confined to the telomere tip, the 166 nt 5′ C-strand signal decreased faster in *yku70Δ* than in *rif2Δ* G1 cells (Figure 4C and 4D). Furthermore, the ∼10 nucleotides decrease in length of the 3′ G-strand was more efficient in *yku70Δ* than in *rif2Δ* G1-arrested cells (Figure 4E). Thus, more resection events are initiated at G1 telomeres in the absence of Yku than in the absence of Rif2. These findings, together with the observation that the shelterin-like proteins still inhibit extensive resection in Yku-lacking cells, suggest that Yku has a major role in preventing initiation of telomere processing, while the shelterin-like proteins are primarily responsible for limiting extensive resection. Accordingly, the concomitant lack of Yku70 and Rif2 showed additive effects on de novo telomere degradation in G1. In fact, both C-strand degradation (Figure 4C and 4D) and generation of r1 resection products (Figure 4E and 4F) occurred more efficiently in G1-arrested *yku70Δ rif2Δ* double mutant cells than in either *yku70Δ* or *rif2Δ* single mutants.

Similar to what we observed after inactivation of Rap1, Rif1 or Rif2 in G2 cells with functional Yku (Figure 2), r1 amounts were higher in *yku70Δ rif2Δ*, *yku70Δ rap1ΔC* and *yku70Δ rif1Δ* cells than in *yku70Δ* single mutant cells after galactose addition in G2 (Figure 4G and 4H), indicating that Rap1, Rif2 and Rif1 inactivation promotes telomere processing in G2 also in the absence of Yku70. The finding that the r1 resection products accumulated with similar kinetics in G2-arrested wild type (Figure 2C) and *yku70Δ* single mutant cells (Figure 4H) further confirms that Yku70 loss does not affect telomere resection in G2.

### Yku70, Rif2, and Rap1 inhibit G-strand overhang generation at native telomeres

**Figure 5:**
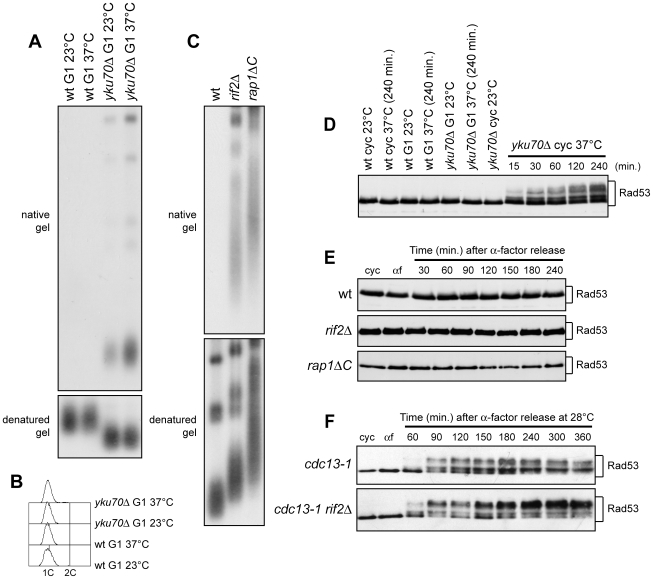
Analysis of single-stranded overhangs at native telomeres. (A,B) G1-arrested (G1) wild type (YLL2599) and otherwise isogenic *yku70Δ* cell cultures were incubated at either 23°C or 37°C for 4 hours in the presence of α-factor. (A) Genomic DNA was digested with XhoI and single-stranded telomere overhangs were visualized by in-gel hybridization (native gel) using an end-labeled C-rich oligonucleotide [31]. The same DNA samples were separated on a 0.8% agarose gel, denatured and hybridized with the end-labeled C-rich oligonucleotide for loading and telomere length control (denatured gel). (B) FACS analysis of DNA content. (C) Genomic DNA prepared from wild type (YLL2599) and otherwise isogenic *rif2Δ* and *rap1ΔC* cell cultures, exponentially growing at 25°C, was digested with XhoI and the single-stranded telomere overhangs were visualized by in-gel hybridization as in (A). (D) Wild type (YLL2599) and otherwise isogenic *yku70Δ* cell cultures exponentially growing (cyc) at 23°C were incubated at 37°C for the indicated time points. G1-arrested wild type and *yku70Δ* cells (G1) were incubated at either 23°C or 37°C for 4 hours. Rad53 was visualized at the indicated times by western analysis with anti-Rad53 antibodies. (E) α-factor arrested wild type (YLL2599) and otherwise isogenic *rif2Δ* and *rap1ΔC* cell cultures were released into the cell cycle at 25°C. Rad53 was visualized as in (D). (F) α-factor-arrested *cdc13-1* and *cdc13-1 rif2Δ* cells were released into the cell cycle at 28°C. Rad53 was visualized as in (D).

The above findings prompted us to investigate whether the key role of Yku, Rif2 and Rap1 in preventing ssDNA generation at de novo telomeres could be extended to native telomeres. As Yku inhibits HO-induced telomere processing specifically in G1, we asked whether Yku70 loss could cause deprotection of native telomeres in G1 cells. To this end, we took advantage of previous data [15] showing that incubation at 37°C of yku70Δ cells causes checkpoint-dependent cell cycle arrest and accumulation of telomeric ssDNA as measured by ssDNA quantitative amplification (QAOS). Thus, we incubated G1-arrested wild type and yku70Δ cells at either 23°C or 37°C for 4 hours in the presence of α-factor (Figure 5B). Genomic DNA was then analyzed by non-denaturing in gel hybridization with a C-rich radiolabeled oligonucleotide detecting the G-rich single-stranded telomere overhangs [31]. As expected, no telomeric ssDNA signals were detectable in G1-arrested wild type cells at either 23°C or 37°C (Figure 5A). In contrast, single-stranded G tail signals appeared in G1-arrested yku70Δ cells even at 23°C, and their intensity increased after incubation at 37°C (Figure 5A), thus highlighting an important role of Yku in protecting native telomeres in G1.

Also Rif2 and Rap1 turned out to inhibit exonucleolytic degradation at native telomeres (Figure 5C). Their role in this process was analyzed in cycling cells, because both proteins protect the HO-induced telomere from degradation in both G1 and G2 cells (Figure 1 and Figure 2). Single-stranded G tails were not detectable in wild type cycling cells, whereas they accumulated in both rif2Δ and rap1ΔC cells, which showed longer native telomeres than wild type, as expected (Figure 5C).

It is well known that a Mec1-dependent DNA damage response is invoked when accumulation of ssDNA at DSBs reaches a certain threshold [9]. We found that G1-arrested yku70Δ cells incubated at either 23°C or 37°C in the presence of α-factor did not show Rad53 electrophoretic mobility shifts that signal Mec1-dependent Rad53 phosphorylation and subsequent checkpoint activation (Figure 5D). Thus, Yku inactivation in G1 does not cause checkpoint activation. By contrast, and consistent with previous data (15), Rad53 phosphorylation was induced when exponentially growing yku70Δ cells were incubated at 37°C (Figure 5D).

We did not observe Rad53 phosphorylation even when G1-arrested *rap1ΔC* and *rif2Δ* cells were released into the cell cycle (Figure 5E), although they accumulated higher amounts of telomeric ssDNA at the HO-induced telomere than *yku70Δ* G1 cells. This lack of checkpoint activation might be due to either limited C-strand resection or general inability to phosphorylate Rad53. We then combined the *rif2Δ* allele with the temperature sensitive *cdc13-1* allele, which is well known to cause C-rich strand degradation and activation of the DNA damage checkpoint after incubation at 37°C [18]–[20] (Figure 5F). When G1-arrested *cdc13-1 rif2Δ* cells were released into the cell cycle at 28°C (semi-permissive temperature for *cdc13-1*), they showed a higher amount of phosphorylated Rad53 than similarly treated *cdc13-1* single mutant cells (Figure 5F). Thus, loss of Rif2 (and possibly of Rap1) enhances the checkpoint response in the presence of partially unprotected telomeres, suggesting that the amount of telomeric ssDNA formation caused by the lack of shelterin-like proteins does not reach the threshold level for the checkpoint response.

### Different nucleases are required for telomeric ssDNA generation in the absence of Yku or shelterin-like proteins

**Figure 6:**
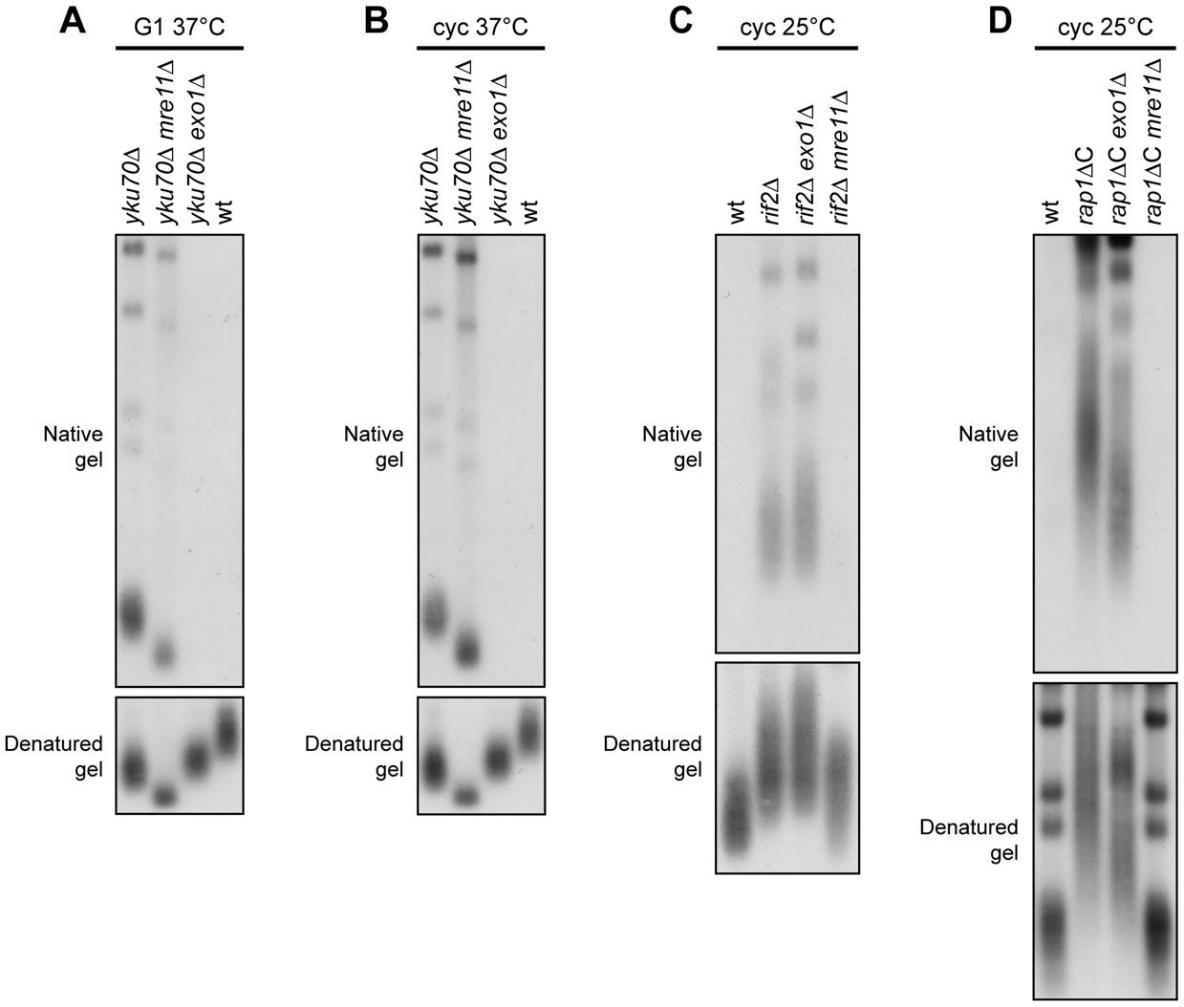
Nuclease requirements for ssDNA generation at native telomeres. (A) G1-arrested cells were incubated at 37°C for 4 hours in the presence of α-factor. Genomic DNA was analyzed as in Figure 5A. (B) Exponentially growing cells were incubated at 37°C for 4 hours. Genomic DNA was analyzed as in Figure 5A. (C,D) Genomic DNA prepared from exponentially growing cells at 25°C was analyzed as in Figure 5A.

As the *yku70Δ*, *rif2Δ* and *rap1ΔC* alleles increased ssDNA generation at native telomeres, we asked which nucleolytic activities were involved in this process. The nuclease Exo1 turned out to be required in both cycling and G1-arrested *yku70Δ* cells. In fact, ssDNA at native telomeres was undetectable in DNA samples prepared from either G1-arrested or exponentially growing *yku70Δ exo1Δ* double mutant cells incubated at 37°C for 4 hours (Figure 6A and 6B). Under the same conditions, *MRE11* deletion only slightly suppressed accumulation of telomeric ssDNA in G1-arrested *yku70Δ* cells (Figure 6A), and did not significantly influence it in cycling *yku70Δ* cells (Figure 6B), indicating that ssDNA generation at native telomeres in the absence of Yku depends primarily on Exo1.

Mre11 was instead required at *rif2Δ* and *rap1ΔC* native telomeres to generate G-rich ssDNA, which was almost completely absent in both *rif2Δ mre11Δ* (Figure 6C) and *rap1ΔC mre11Δ* cycling cells (Figure 6D). By contrast, *EXO1* deletion did not affect the same process in *rif2Δ* and *rap1ΔC* cycling cells (Figure 6C and 6D). Thus, native telomere nucleolytic degradation that is normally inhibited by Rif2 and Rap1 is mainly Mre11-dependent.

**Figure 7:**
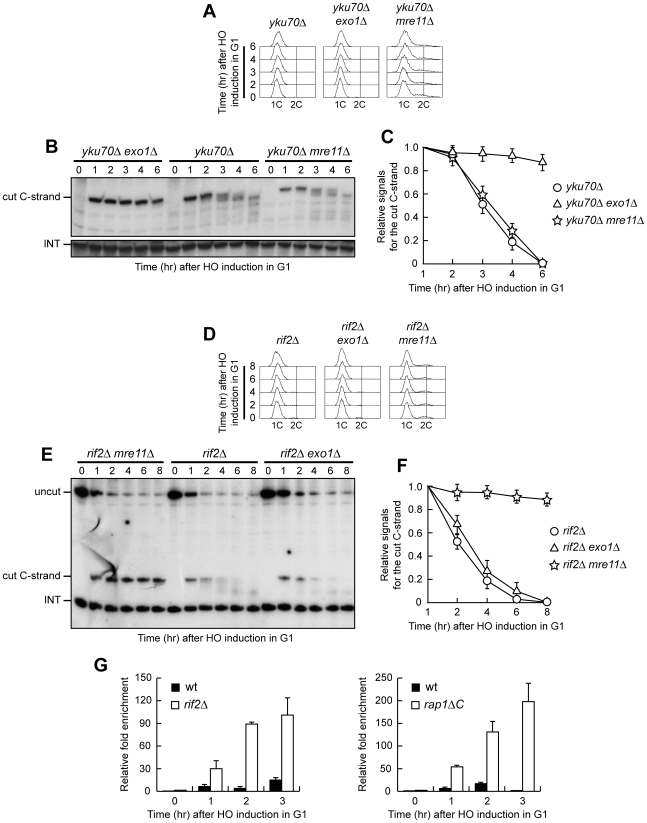
Nuclease requirements for ssDNA generation at a de novo telomere. (A–C) HO expression was induced at time zero by galactose addition to α-factor-arrested *yku70Δ*, *yku70Δ exo1Δ* and *yku70Δ mre11Δ* cell cultures that were then kept arrested in G1. (A) FACS analysis of DNA content. (B) RsaI-digested genomic DNA was hybridized with probe A as in Figure 3E. (C) Densitometric analysis. Plotted values are the mean value ±SD from three independent experiments as in (B). (D–F) HO expression was induced at time zero by galactose addition to α-factor-arrested *rif2Δ*, *rif2Δ exo1Δ* and *rif2Δ mre11Δ* cell cultures that were then kept arrested in G1. (D) FACS analysis of DNA content. (E) RsaI- and EcoRV-digested genomic DNA was hybridized with probe A as described in Figure 1C. (F) Densitometric analysis. Plotted values are the mean value ±SD from three independent experiments as in (E). (G) HO expression was induced at time zero by galactose addition to α-factor-arrested wild type, *rif2Δ* and *rap1ΔC* cells, all expressing a fully functional *MRE11-MYC* tagged allele. Cells were then kept arrested in G1 and chromatin samples taken at different times after HO induction were immunoprecipitated with anti-Myc antibody. Coimmunoprecipitated DNA was analyzed by quantitative real-time PCR (qPCR) using primer pairs located at the nontelomeric *ARO1* fragment of chromosome IV (CON) and 640 bp proximal to the HO site (TEL), respectively. Data are expressed as relative fold enrichment of TEL over CON signal after normalization to input signals for each primer set. The data presented are the mean of those obtained in three independent experiments. Error bars indicate s. d.

The absence of Mre11 leads to telomere shortening in *yku70Δ*, *rif2Δ* and *rap1ΔC* cells (Figure 6), likely because it prevents loading of the Tel1 kinase, which in turn allows recruitment of the Est1 telomerase subunit by phosphorylating Cdc13 [32]–[34]. In order to rule out possible artefacts caused by telomere structure alterations, we analyzed the effects of the *mre11Δ* and *exo1Δ* alleles also at the newly created HO-induced telomere in G1 cells that cannot elongate this telomere due to the low Cdk1 activity. Similar to what we observed at native telomeres, 5′ C-strand degradation in G1-arrested *yku70Δ* cells was abolished in the absence of Exo1, whereas it occurred in *yku70Δ mre11Δ* cells (Figure 7A–7C). Conversely, the lack of Exo1 did not affect 5′ C-strand degradation in G1-arrested *rif2Δ* cells, where degradation of the same strand was instead abolished in the absence of Mre11 (Figure 7D–7F). Unfortunately, we were unable to synchronize *rap1ΔC mre11Δ* and *rap1ΔC exo1Δ* cell cultures due to their growth defects (data not shown). Altogether, these data indicate that Exo1 is primarily responsible for telomeric DNA degradation in the absence of Yku70, whereas the same process is mainly Mre11-dependent in *rif2Δ* and *rap1ΔC* cells, suggesting that Yku and shelterin-like proteins specifically prevent the action of different nucleases.

### Rif2 and Rap1 inhibit Mre11 association at a de novo telomere in G1

Our data indicate that Mre11 plays a key role in telomeric ssDNA generation in the absence of Rif2 or Rap1, and Rif2 has been shown to regulate MRX recruitment at telomeres in cycling cells by inhibiting Tel1 association at telomeric ends [35]. We then monitored Mre11 recruitment at the HO-induced telomere in G1-arrested wild type, *rif2Δ* and *rap1ΔC* cells carrying a fully functional *MYC*-tagged *MRE11* allele. Sheared chromatin from formaldehyde cross-linked cell samples taken at different time points after galactose addition was immunoprecipitated with anti-Myc antibodies. Quantitative real-time polymerase chain reaction (qPCR) was then used to monitor coimmunoprecipitation of a DNA fragment located 640 bp centromere-proximal to the HO site (TEL) and of a nontelomeric *ARO1* fragment (CON). The TEL/CON ratio, which was used to measure Mre11 association with the HO-induced telomere, was much higher in both *rif2Δ* and *rap1ΔC* cells than in wild type (Figure 7G), indicating that Rif2 and Rap1 prevent Mre11 association at telomeric ends in G1. This finding, together with the observation that Mre11 is required to generate telomeric ssDNA in the absence of Rif2 or Rap1, suggests that Rif2 and Rap1 might inhibit telomere processing by preventing Mre11 binding.

## Discussion

Previous studies have shown that processing of *S. cerevisiae* telomeres is less efficient in G1 than in G2/M and Cdks are crucial for this difference [6], [7]. This work identifies the shelterin-like proteins Rap1, Rif1 and Rif2, as well as Yku, as other important players in the regulation of this process, where they inhibit nucleolytic telomere degradation. In particular, lack of Rif1, Rif2 or C-terminus of Rap1 promote C-rich strand degradation at an HO-derived telomere in G1 and enhance it in G2. Moreover, cycling cells devoid of Rif2 or Rap1 C-terminus display accumulation of ssDNA also at native telomeres. Thus, all these shelterin-like proteins inhibit nucleolytic degradation at telomeres, with Rap1 and Rif2 showing the strongest effects. Consistent with our finding, end processing and Mre11 binding have been shown to be reduced at an HO-induced telomere with 250 bp TG tracts compared to one with 81 bp TG tracts [36], which likely bind a smaller number of Rap1-Rif1-Rif2 complexes than the former [22], [23]. Interestingly, ssDNA generation at both native and HO-induced telomeres is increased to the same extent in *rif2Δ* and *rap1ΔC* cells, suggesting that the effect exerted by Rap1 is likely mediated by Rif2. In fact, Rap1 recruits Rif2 to the TG tracts through its C-terminal domain [26]. On the other hand, also Rif1 is recruited by Rap1 to TG tracts [24], [25], but Rif1 loss has a minor effect on C-strand resection, indicating different functions for Rif1 and Rif2 in inhibiting nucleolytic telomere processing. Similarly, Rif2, but not Rif1, prevents telomeric fusions by NHEJ [21].

Also Yku has a role in inhibiting telomere resection, but it acts specifically in G1. In fact, ssDNA generation at both HO-induced and native telomeres is increased in G1-arrested *yku70Δ* cells compared to wild type, whereas no significant differences are observed in G2/M. The Yku-mediated inhibitory effect on telomeric processing is independent on Yku role in NHEJ, as Dnl4 loss does not promote ssDNA generation at the HO-induced telomere in G1, unlike at intrachromosomal DSBs [9], [30]. This finding is consistent with the observation that NHEJ is inhibited at telomeres [37], possibly because its components are excluded from telomeric ends. Interestingly, resection at the HO-induced telomere in G1-arrested *yku70Δ* cells does not proceed beyond 166 bp from the HO site, suggesting that either the rate or the processivity of resection is reduced in G1 compared to G2/M in the absence of Yku. It is noteworthy that this limited processing is due to the inhibitory action of Rap1, Rif1 and Rif2, as their inactivation allows extensive resection not only in wild type but also in *yku70Δ* G1 cells.

Although C-strand degradation in the absence of Yku is restricted to the regions closest to the telomeric tip, this degradation is more efficient in G1-arrested *yku70Δ* cells than in *rif2Δ* cells. This observation, together with the finding that the shelterin-like proteins limit extensive resection in Yku-lacking cells, suggests that Yku is mainly involved in inhibiting initiation, whereas Rif1, Rif2 and Rap1 act primarily by limiting extensive resection. Consistent with the different inhibitory functions of Yku and shelterin-like proteins, the concomitant lack of Yku and Rif2 has additive effects on de novo telomere degradation in G1. In fact, both C-strand degradation and generation of r1 resection products occur more efficiently in G1 *yku70Δ rif2Δ* double mutant cells than in *rif2Δ* and *yku70Δ* single mutants.

It is worth pointing out that telomere processing in the absence of Yku, Rif2, Rap1 or Rif1 takes place in G1 independently of the low Cdk1 activity. As DSB resection is not completely abolished in G1 [5], [30], the Cdk1 role might be simply to potentiate the resection machineries, thus explaining why Cdk1 requirement for telomere resection can be bypassed by inactivation of negative regulators of this process.

Interestingly, even the 3′ G strand of the HO-induced telomere decreases ∼10 nucleotides in length and this limited degradation seems to correlate with the ability to initiate 5′-3′ processing. This phenomenon is reminiscent of the removal of the 3′ overhangs from uncapped telomeres by the human nucleotide excision repair endonuclease ERCC1/XPF [38]. Although the physiological significance of the 3′ G strand shortening is unknown, removal of these nucleotides might facilitate telomerase RNA annealing to its template.

The resection extent at the HO-induced telomere is higher in *rif2Δ* and *rap1ΔC* cycling cells than in *yku70Δ* G1 cells, but ssDNA at native telomeres does not elicit the DNA damage checkpoint in any of these mutant cells, suggesting that other mechanisms might prevent a DNA damage response at telomeres. One possibility is that the ssDNA accumulated in the absence of Yku or the shelterin-like proteins is still covered by Cdc13, which has been shown to inhibit Mec1 association to DNA ends [12]. Consistent with this hypothesis, the lack of Rif2 enhances checkpoint activation in cells crippled for Cdc13 activity (Figure 5F). Alternatively, or in addition, as Mec1 is the main responder to DSBs in yeast and its activation needs ssDNA [39], the amount of telomeric ssDNA in these cells may be insufficient to elicit a checkpoint response. In mammalian cells, loss of the shelterin protein TRF2 leads to ATM-dependent DNA damage response that does not require extensive degradation of the telomeric 5′ strand [40]. The knowledge that the ATM yeast ortholog, Tel1, has a very minor role in the checkpoint response to DSBs compared to Mec1 [41] might explain this difference between yeast and mammals in the response to telomere alterations.

**Figure 8:**
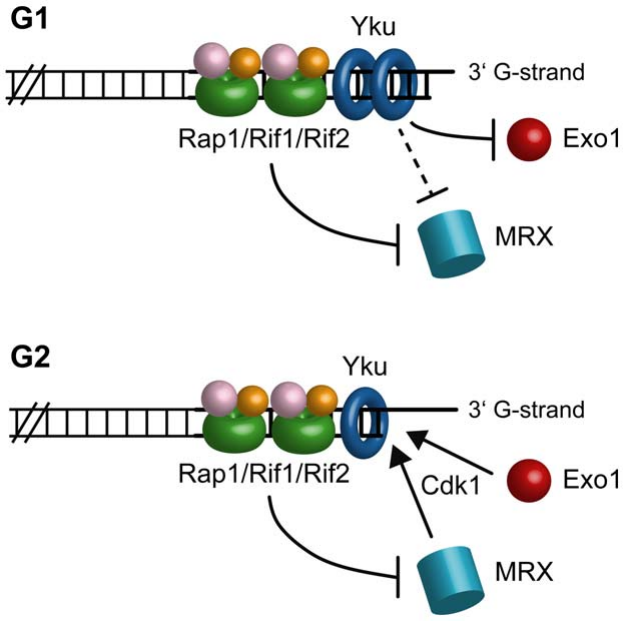
A working model for limiting DNA degradation at telomeres. In G1, Yku protects telomeres from Exo1, while Rap1, Rif1 and Rif2 mainly act by preventing MRX access. As MRX action is still inhibited by Rap1, Rif1 and Rif2 in *yku70Δ* G1 cells, Yku might protect G1 telomeres also from MRX. In G2, only Rap1 and Rif2 still exert their inhibitory effects on telomere processing. Telomere resection can take place in G2 because Yku does not exert its inhibitory effect and Cdk1 activity potentiates nuclease actions.

The inhibitory actions of Yku and shelterin-like proteins seem to target different nucleases. In fact, Exo1 appears to be important for telomeric ssDNA generation at both native and HO-induced telomeres in *yku70Δ* G1 cells, suggesting that Yku might hide the telomeric ends from Exo1 association. By contrast, telomeric ssDNA generation in both *rif2Δ* and *rap1ΔC* cells depends primarily on Mre11, whose recruitment in G1 to the HO-induced telomere is enhanced in *rif2Δ* and *rap1ΔC* cells. Thus, while Yku protects telomeres towards Exo1 in G1, Rap1 and Rif2 likely prevent telomere processing by inhibiting loading of the MRX complex onto telomeric ends in both G1 and G2 (Figure 8). However, we cannot exclude that Yku might protect G1 telomeres also from MRX (Figure 8), as it has been observed at intrachromosomal DSB [30], because MRX action in *yku70Δ* G1 cells is anyhow inhibited by Rap1, Rif1 and Rif2. The nuclease responsible for telomere processing in the absence of the shelterin-like proteins might be MRX itself and/or the endonuclease Sae2, which was shown to act in concert with MRX in telomere processing [4]. As some MRX association at the HO-induced telomere can be detected in wild type G1 cells, Rap1 and Rif2 might impair 5′-end resection also by inhibiting MRX/Sae2 activity besides its association to DNA. In any case, telomere processing can take place in G2, likely because Cdk1 activity potentiates the resection machinery and Yku does not exert its inhibitory effect in this cell cycle phase (Figure 8).

It is noteworthy that Exo1 is a key factor for ssDNA generation at telomeres in Yku-lacking cells, while it plays only a minor role in doing so at intrachromosomal DSBs, where resection in *yku70Δ* mutant cells is primarily MRX-dependent [30] (our unpublished data). As Rif2 and Rap1 inhibit telomere degradation even in the absence of Yku, their presence at telomeres might block MRX access, thus explaining the different requirements of nuclease activities at DSBs versus telomeres in the absence of Yku.

Telomere protecting mechanisms are particularly important to prevent illegitimate repair/recombination, whose outcomes at telomeres can range from the generation of chromosomal abnormalities, general hallmarks for human cancer cells, to permanent cell cycle arrest and cell death. Altogether, this work increases our knowledge of this complex regulation, as it highlights a role of evolutionarily conserved proteins in protecting chromosome ends during different cell cycle phases by preventing the action of different nucleases.

## Materials and Methods

### Strains and plasmids

Strain genotypes are listed in Table S1. The strains used for monitoring telomere resection at the HO-induced telomere and HO-induced DSB were derivatives of strains UCC5913 and RMY169, respectively, kindly provided by D. Gottschling (Fred Hutchinson Cancer Research Center, USA) and T. Weinert (University of Arizona, USA). Strain RMY169 was created by replacing the *ADE2-TG* cassette of strain UCC5913 with the *TRP1* gene [11]. In order to allow an efficient and persistent G1 arrest, all strains carried the deletion of the *BAR1* gene, encoding a protease that degrades the mating pheromone α-factor. The *cdc13-1* mutant was kindly provided by D. Lydall (University of Newcastle, UK.). The plasmid pM585, carrying the *rap1Δ670-807* allele, was kindly provided by D. Shore (University of Geneva, Switzerland). Cells were grown in YEP medium (1% yeast extract, 2% bactopeptone, 50 mg/l adenine) supplemented with 2% glucose (YEPD) or 2% raffinose (YEP+raf) or 2% raffinose and 2% galactose (YEP+raf+gal). Unless otherwise stated, all the experiments were carried out at the temperature of 25°C.

### Western blot analysis

Protein extracts were prepared by TCA precipitation as described in [42]. Rad53 was detected using anti-Rad53 polyclonal antibodies kindly provided by J. Diffley (Clare Hall, London, UK). Secondary antibodies were purchased from Amersham and proteins were visualized by an enhanced chemiluminescence system according to the manufacturer.

### Resection assay

Visualization of the single-stranded overhangs at native telomeres was done as described [31]. The same DNA samples were separated on a 0.8% agarose gel, denatured and hybridized with the end-labeled C-rich oligonucleotide for loading control. To monitor resection at the HO-derived telomeres, RsaI- and EcoRV-digested genomic DNA was subjected to denaturing polyacrilammide gel electrophoresis and then hybridized with the single-stranded riboprobes A or B, which anneal to the 5′ C-strand or the 3′ G-strand, respectively, to a site located 212 nt from the HO cutting site. Resection of the C-rich strand in Figure 3E and Figure 4C was monitored by hybridizing RsaI-digested genomic DNA with riboprobe A. To monitor resection of the 5′-strand at the HO-induced DSB, EcoRV-digested genomic DNA was hybridized with a single-stranded riboprobe, which anneal to the 5′-strand to a site located 215 nt from the HO cutting site. For quantitative analysis of C-strand and G-strand signals, the ratios between the intensities of ssDNA and loading control bands were calculated by using the NIH image program.

### ChIP analysis

ChIP analysis was performed as described [43]. After exposure to formaldehyde, chromatin samples were immunoprecipitated with anti-Myc antibody. Quantification of immunoprecipitated DNA was achieved by qPCR on a Biorad MiniOpticon using primer pairs located at the nontelomeric *ARO1* fragment of chromosome IV (CON) and 640 bp centromere-proximal to the HO cutting site (TEL) and normalized to input signal for each primer set; data are expressed as the fold enrichment of TEL over the amount of CON in the immunoprecipitates.

## Supporting Information

Table S1 *Saccharomyces cerevisiae* strains used in this study.
(0.05 MB DOC)
