# Supplementary material for: Shelterin-Like Proteins and Yku Inhibit Nucleolytic Processing of Saccharomyces cerevisiae Telomeres
Source: PLoS Genet. 2010 May 27;6(5):e1000966. doi: 10.1371/journal.pgen.1000966 (PMC2877729; doi:10.1371/journal.pgen.1000966)
Supplement: Table S1 — Saccharomyces cerevisiae strains used in this study. (0.05 MB DOC) [file pgen.1000966.s001.doc]

# Supporting Information

**Table S1.** *Saccharomyces cerevisiae* strains used in this study.

| Strain | Relevant genotype | Source or Reference |
| --- | --- | --- |
| UCC5913 | *MATa-inc ade2-101 lys2-801 his3-Δ200 trp1-Δ63 ura3-52 leu2-Δ1::GAL1-HO-LEU2 VII-L::ADE2-TG(1-3)-HO site-LYS2* | 1 |
| RMY169 | *MATa-inc ade2-101 lys2-801 his3-Δ200 trp1-Δ63 ura3-52 leu2-Δ1::GAL1-HO-LEU2 VII-L::TRP1-HO site-LYS2* | 2 |
| YLL2554 | UCC5913 MRE11-18MYC::TRP1 | This study |
| YLL2599 | UCC5913 *bar1Δ::HPHMX* | This study |
| YLL2600 | RMY169 *bar1Δ::KANMX4* | This study |
| YLL2606 | RMY169 *yku70Δ::URA3 bar1Δ::HPHMX* | This study |
| YLL2607 | RMY169 *dnl4Δ::NATMX bar1Δ::HPHMX* | This study |
| YLL2612 | UCC5913 *yku70Δ::URA3 bar1Δ::HPHMX* | This study |
| YLL2613 | UCC5913 *dnl4Δ::NATMX bar1Δ::HPHMX* | This study |
| YLL2646 | UCC5913 *yku70Δ::URA3 rif1Δ::NATMX bar1Δ::HPHMX* | This study |
| YLL2647 | UCC5913 *yku70Δ::URA3 rif2Δ::NATMX* *bar1Δ::HPHMX* | This study |
| YLL2649 | UCC5913 *rif1Δ::NATMX bar1Δ::HPHMX* | This study |
| YLL2650 | UCC5913 *rif2Δ::NATMX bar1Δ::HPHMX* | This study |
| YLL2651 | UCC5913 *rap1Δ::KANMX4* [*CEN-HIS3-rap1Δ670-807*] *bar1Δ::HPHMX* | This study |
| YLL2655 | UCC5913 *yku70Δ::URA3 rap1Δ::KANMX4* [*CEN-HIS3-rap1Δ670-807*] *bar1Δ::HPHMX* | This study |
| YLL2670 | UCC5913 MRE11-18MYC::TRP1 *bar1Δ::HPHMX* | This study |
| YLL2672 | UCC5913 MRE11-18MYC::TRP1 *rap1Δ::KANMX4* [*CEN-HIS3-rap1Δ670-807*] *bar1Δ::HPHMX* | This study |
| YLL2694 | UCC5913 MRE11-18MYC::TRP1 *rif2Δ::NATMX* | This study |
| YLL2725 | UCC5913 *rif2Δ::NATMX mre11Δ::KANMX4 bar1Δ::HPHMX* | This study |
| YLL2728 | UCC5913 *yku70Δ::URA3* *exo1Δ::NATMX bar1Δ::HPHMX* | This study |
| YLL2730 | UCC5913 *yku70Δ::URA3 mre11Δ::NATMX bar1Δ::HPHMX* | This study |
| YLL2731 | UCC5913 *rif2Δ::NATMX exo1Δ::URA3 bar1Δ::HPHMX* | This study |
| YLL2733 | UCC5913 *rap1Δ::KANMX4* [*CEN-HIS3-rap1Δ670-807*] *exo1Δ::URA3 bar1Δ::HPHMX* | This study |
| YLL2736 | UCC5913 *rap1Δ::KANMX4* [*CEN-HIS3-rap1Δ670-807*] *mre11Δ::NATMX bar1Δ::HPHMX* | This study |
| K699 | *MATa* *ade2-1 can1-100 his3-11,15 leu2-3,112 trp1-1 ura3-1 rad5-535* | This study |
| DMP5108/19A | K699 *cdc13-1* | This study |
| DMP5108/20A | K699 *cdc13-1 rif2Δ::KANMX4* | This study |

Plasmids are indicated by brackets

**References**

1. Diede SJ, Gottschling DE (2001) Exonuclease activity is required for sequence addition and Cdc13p loading at a de novo telomere. Curr Biol 11: 1336-1340.

2. Michelson RJ, Rosenstein S, Weinert T (2005) A telomeric repeat sequence adjacent to a DNA double-stranded break produces an anticheckpoint. Genes Dev 19: 2546-2559.
